# Salvage total hip arthroplasty after failed internal fixation for proximal femur and acetabular fractures

**DOI:** 10.1186/s13018-023-03519-9

**Published:** 2023-01-17

**Authors:** Ching-Chieh Hung, Kuan-Hsiang Chen, Chih-Wei Chang, Yi-Chen Chen, Ta-Wei Tai

**Affiliations:** 1 Departments of Orthopedics, National Cheng Kung University Hospital, College of Medicine, National Cheng Kung University, 138 Sheng-Li Road, Tainan, 70428 Taiwan; 2 Departments of Nursing, National Cheng Kung University Hospital, College of Medicine, National Cheng Kung University, Tainan, Taiwan; 3 Skeleton Materials and Bio-compatibility Core Lab, Research Center of Clinical Medicine, National Cheng Kung University Hospital, College of Medicine, National Cheng Kung University, Tainan, Taiwan

**Keywords:** Total hip arthroplasty, Failed internal fixation, Revision total hip arthroplasty, Hip fractures, Acetabular fractures

## Abstract

**Background:**

Total hip arthroplasty (THA) is the treatment of choice for posttraumatic arthritis with failed internal fixation for hip fractures. However, the postoperative prognosis is not clear.

**Questions/purposes:**

The primary aim of the study is to report the postoperative outcome, prognosis, and complication rates of total hip arthroplasty in posttraumatic hip arthritis after failed internal fixation of fractures around the hip. The secondary aim of the study is to report results among different fracture types around the hip.

**Patients and methods:**

We enrolled salvage THA patients after failed internal fixation of fractures around the hip and matched control patients undergoing primary THA for hip osteoarthritis. Subgroup analysis was performed to compare the postoperative outcomes, prognosis, and complication rates of salvage THA in posttraumatic hip arthritis after failed internal fixation of different fracture types around the hip.

**Results:**

A total of 315 THAs (105 salvage THAs and 210 primary THAs) were analyzed. Patients with salvage THA had a longer operative time, lower postoperative hemoglobin (Hb) level, more Hb drop (2.2 ± 1.4 vs. 1.7 ± 1.2 gm/dl, *p* = 0.002), and delayed ambulation. The salvage THA group also had a higher dislocation rate within 2 months after salvage THA (9.5% vs. 1.9%, *p* = 0.002), reoperation rate (10.5% vs. 3.8%, *p* = 0.019, including debridement, open and closed reduction under sedation, revision surgery, surgical fixation for periprosthetic fractures), and revision rate (5.7% vs. 0.5%, *p* = 0.003) than patients undergoing primary THA. Patients who had failed fixation for acetabular fractures were younger and tended to recover well. Patients with previous intertrochanteric fracture had the longest operative time, more hip pain (83.8%, *p* = 0.022) and more complications.

**Conclusion:**

Salvage THA in posttraumatic hip arthritis after failed internal fixation required a longer operative time and led to more blood loss and postoperative complications. The dislocation, reoperation, and revision rates after salvage THA were higher than those after primary THA. Patients with salvage THA after failed internal fixation for intertrochanteric fractures were the most susceptible to more complications compared to those with femoral neck fracture or acetabular fracture.

*Level of Evidence* level III

**Supplementary Information:**

The online version contains supplementary material available at 10.1186/s13018-023-03519-9.

## Introduction

Total hip arthroplasty (THA) is one of the most common, successful, and cost-effective surgeries that relieves refractory pain and restores mobility around the hip joint [1, 2]. Indications for THA include primary osteoarthritis, ankylosing spondylitis, osteonecrosis, rheumatoid arthritis, and posttraumatic arthritis [2–4]. Compared with THA for other indications, salvage THA for posttraumatic arthritis is more complicated and technically challenging [5].

Femoral neck and intertrochanteric fractures account for most proximal femoral fractures [6]. The management of internal fixation for proximal femoral or hip fractures can fail due to dislocation, loss of fixation, femoral head osteonecrosis, periprosthetic fracture, surgical site infection, or symptomatic deterioration [7–13]. An acetabular fracture is an intra-articular fracture often requiring open reduction and internal fixation. Posttraumatic hip arthritis commonly develops afterward. Salvage THA is indicated in patients with end-stage disease.

THA is a salvage treatment of choice for failure of internal fixation for both proximal femoral and acetabular fractures [13]. However, it may yield more complications than THA for primary osteoarthritis [9–11, 14–18]. Some previous studies reported more blood loss and operative time in THA for posttraumatic arthritis [9, 19]. The other series showed compatible postoperative outcomes for both primary osteoarthritis and posttraumatic arthritis [10, 20]. There is seldom a head-to-head comparison of outcomes between primary osteoarthritis and posttraumatic arthritis in the literature. The outcomes of THA after different types of fractures and fixations are still unclear [10–12, 14–18, 21–24].

The purpose of this study was to identify and compare the prognosis and outcomes of patients who received salvage THA after failed internal fixation for fractures around the hip and primary THA for primary osteoarthritis. We also compared the outcomes of salvage THA after different types of internal fixation for each subgroup.

## Materials and methods

This was a single-center analysis in a tertiary medical center. All medical records were collected with standardized collection tools. The study was approved by the review board of our institute.

### Patient information and clinical assessment

We enrolled patients who underwent salvage THA after failed internal fixation from 2013 to 2019. Patient recruitment was based on the following inclusion criteria: age ≥ 18 years, plain film-based diagnosis clearly showing failure of previous internal fixation for fractures (femoral neck fracture, intertrochanteric fracture, and acetabular fracture), with destruction of hip joints caused by posttraumatic arthritis, osteonecrosis, or nonunion. The exclusion criteria included age < 18 years, previous treatment with hemiarthroplasty, pathological fractures, and extremely large acetabular bone defects that could not be reconstructed with a multihole revision acetabular cup (severer than Paprosky classification type IIB).

The other 1:2 age- and sex-matched cohort of patients with THA for primary osteoarthritis was enrolled as a control group. The sample size was calculated by G*Power software version 3.1.9.7 (Heinrich-Heine-Universität Düsseldorf, Düsseldorf, Germany). All patients involved had suffered from hip pain and symptoms, including limping gait, limited activity, and difficulty accomplishing activities of daily living.

### Data collection and outcome measurement

We collected demographic data, including age, sex, follow-up years, and medical comorbidities. The following perioperative information was recorded: operative time, preoperative/postoperative hemoglobin (Hb) level, decline in hemoglobin level, postoperative ambulatory status, blood transfusion, and length of stay. The postoperative day to ambulate was defined as walking with a walker for at least 10 m without others' assistance. Postoperative complications were also recorded. These included persistent hip pain, prosthetic dislocation, superficial or deep infection, poor wound healing, reoperation, and revision THA. Deep infection was defined as surgical debridement. Superficial infection indicated that the infection was successfully treated by wound dressing and medication.

For subgroup analysis, patients undergoing salvage THA were further divided into 3 subgroups based on fracture type: femoral neck fracture, intertrochanteric fracture, and acetabulum fracture. We also performed another subgroup analysis based on different types of internal fixation.

### Surgical methods and postoperative protocol

Patients received either general or local anesthesia depending on the anesthesiologist’s evaluation. All THA procedures were performed by experienced orthopedic surgeons. Previous fixation implants for proximal femoral fracture were removed. Previous fixation implants for acetabular fracture were removed if necessary. Then, capsular fibrosis, hypertrophic synovium, osteophytes, and destroyed cartilage around the fractures were fully removed to approach the surgical fields for THA. Selection of the THA prosthesis depended on the patient’s anatomy, degree of acetabular and femoral bone defects, and bone quality according to intraoperative evaluation after implant removal. Multihole acetabular cups were used for cases with previous acetabular fractures. If the stem could not be stabilized by the press-fit technique, a cemented stem was used. Long stems were selected when the femoral calcars or subtrochanteric structure was involved. Postoperative care and rehabilitation were provided according to routine postoperative protocols. We encouraged all patients to perform postoperative exercise as soon as possible. The patients were allowed to perform weight-bearing standing and ambulation with a walker as tolerated later in the operative day. We arranged the same standard rehabilitation program for both groups. Physical therapists provided instructions for ankle pumping, muscle stretching and strengthening, position transition, weight bearing, and ambulation.

### Outcome evaluation

Patients received follow-up clinically and radiologically at 2 weeks, 1 month, 3 months, 6 months, and 1 year and each year thereafter postoperatively in the outpatient clinic. The orthopedic nurse practitioners evaluated the joint specific, patient-reported outcome of patients one year postoperatively, and the Oxford Hip Score was recorded.

### Statistical analysis

SPSS version 17.0 (IBM, NY, USA) statistical software was used for the data analysis. Categorical variables were analyzed by Fisher’s exact test and displayed as counts and proportions. Continuous variables are presented as the mean and standard deviation (SD) and were assessed using Student’s t test. Kaplan–Meier survival curves and log-rank tests were used to analyze survival and cumulative incidence. In subgroup analysis, one-way ANOVA was used for continuous variables with a Gaussian distribution, and the Kruskal–Wallis test was used for those with a non-Gaussian distribution. Categorical variables were analyzed by the Chi-square test. A *p* value < 0.05 was considered statistically significant.

## Results

A total of 315 patients who underwent THA were included in this study. We enrolled 105 patients who underwent THA after fixation failure (the salvage THA group) and 210 age- and sex-matched patients who underwent THA for primary osteoarthritis. The patients consisted of 165 men and 150 women with an overall mean age of 62.8 ± 17.7 years (ranging from 18 to 91 years) and a median follow-up of 1.6 ± 1.7 years (ranging from 9 days to 8 years) (Table 1). Follow-up years were significantly longer in the salvage THA group (Table 1).

**Table 1:** Basic characteristics and medical comorbidities of patients undergoing THA

	THA after fixation failure	Primary THA	*p* value
No. of patients	105	210	–
Sex (male/female)	(55/50)	(110/100)	1.00
Age	62.8 (17.9)	62.8 (17.7)	0.987
Follow-up years	1.9 (1.7)	1.4 (1.6)	0.004*
Medical history			
Diabetes mellitus	20 (19%)	24 (11.4%)	0.066
Hypertension	46 (43.8%)	95 (45.2%)	0.81
Coronary artery disease	4 (3.8%)	12 (5.7%)	0.468
Chronic kidney disease	10 (9.5%)	13 (6.2%)	0.284
Cerebrovascular accident	8 (7.6%)	2 (1%)	0.001*
Rheumatoid arthritis	3 (2.9%)	4 (1.9%)	0.589
Peptic ulcer disease	10 (9.5%)	13 (6.2%)	0.284
Obesity	3 (2.9%)	2 (1%)	0.202
Systemic lupus erythematosus	1 (1%)	4 (1.9%)	0.524
Malignancy (cancer)	8 (7.6%)	19 (9%)	0.669
Death during follow-up period	4 (3.8%)	5 (2.4%)	0.473

Age and follow-up years were expressed as the mean (standard deviation)

Medical history was expressed as a number (percentage)

Unpaired *t* test for age and follow-up years

Fisher’s test for all medical history data

*p < 0.05

The mean operative time of the salvage THA group was 132.5 ± 51.4 min, which was significantly longer than that of the primary THA group (87.6 ± 34.3 min, *p* < 0.001) (Table 2). The preoperative Hb levels were similar in both groups. However, the postoperative Hb level was significantly lower in the salvage THA group (10.3 ± 1.4 vs. 11.1 ± 1.6 gm/dl, *p* < 0.001). The drop in hemoglobin levels was also significantly greater in the salvage THA group (2.2 ± 1.4 vs. 1.7 ± 1.2 gm/dl, *p* = 0.002). There was no difference between the 2 groups regarding the blood transfusion rate and the amount of blood transfused. The postoperative day to ambulate was significantly longer in the salvage THA group than in the primary THA group (2.1 ± 0.9 vs. 1.7 ± 1.0 days, *p* = 0.001). The length of stay was similar between the two groups.

**Table 2:** Prognosis and outcome of patients undergoing THA

	THA after fixation failure (*n* = 105)	Primary THA (*n* = 210)	*p* value
Operative time (minute)	132.5 (51.4)	87.6 (34.3)	< 0.001*
Hemoglobin level			
Preoperative	12.5 (1.8)	12.8 (1.9)	0.159
Postoperative	10.3 (1.4)	11.1 (1.6)	< 0.001*
Drop in hemoglobin level	2.2 (1.4)	1.7 (1.2)	0.002*
Blood transfusion	14 (13.3%)	24 (11.4%)	0.625
Amount of blood transfusion (unit)	0.63 (3.1)	0.52 (2.9)	0.754
Postoperative day to ambulate	2.1 (0.9)	1.7 (1.0)	0.001*
Length of stay (day)	7.3 (1.3)	7.1 (2.3)	0.429

Operative time, hemoglobin level, drop in hemoglobin level, postoperative day to ambulate, amount of blood transfusion, and length of stay were expressed as the mean (standard deviation)

Blood transfusion was expressed as a number (percentage)

Postoperative day to ambulate was defined as walking with a walker for at least 10 m without others' assistance

Unpaired *t* test for operative time, hemoglobin level, drop in hemoglobin level, postoperative day to ambulate, amount of blood transfusion, and length of stay

Fisher’s test for blood transfusion

*p < 0.05

**Fig. 1:**
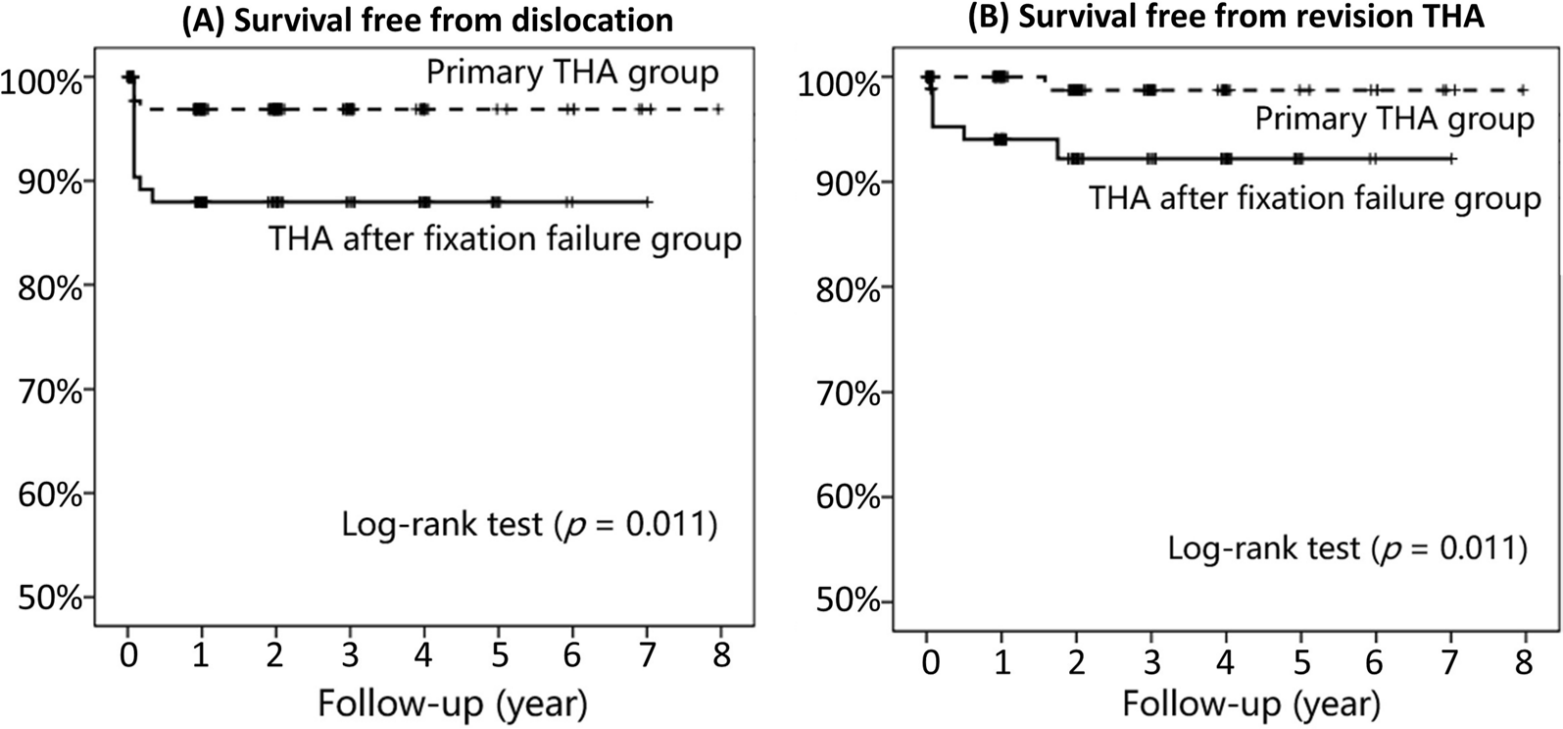
Kaplan–Meier survival curves from **A** prosthetic dislocations and **B** revision of total hip arthroplasty (THA)

The incidence of prosthetic dislocation was significantly higher in the salvage THA group (9.5%, 10/105) than in the primary THA group (1.9%, 4/210) (*p* = 0.002) (Table 3). Most dislocations (9/10 in the salvage THA group and all 4 patients in the primary THA group) occurred within the first 2 months after THA (Fig. 1A). The causes reported by patients themselves were deep flexion of the hip > 90 degrees (9 patients), pivot rotation (7 patients), and slipping down (3 patients). All patients with prosthetic dislocation underwent surgical intervention, including revision THA for prosthetic loosening (6 patients in the salvage THA group) and open reduction and tightening of the soft tissue envelope (4 patients in each group).

**Table 3:** Complications after total hip arthroplasty in the two groups

	THA after fixation failure (*n* = 105) (%)	Primary THA (*n* = 210) (%)	*p* value
Persistent hip pain	75 (71.4)	146 (69.5)	0.728
Prosthetic dislocation	10 (9.5)	4 (1.9)	0.002*
Wound poor healing	3 (2.9)	6 (2.9)	1
Superficial infection	1 (1)	0 (0)	0.157
Deep infection	4 (3.8)	3 (1.4)	0.177
Reoperation	11 (10.5)	8 (3.8)	0.019*
THA revision	6 (5.7)	1 (0.5)	0.003*

Persistent hip pain, prosthetic dislocation, superficial infection, deep infection, poor wound healing, reoperation, readmission, and THA revision were expressed as numbers (percentages)

Persistent hip pain was defined as still having significant hip pain 3 months after THA that needed medication for pain control

Fisher’s test for all data

^*^p < 0.05

Patients who underwent THA after fixation failure tended to have a higher incidence of deep infection (3.8% vs. 1.4%), but the difference did not reach statistical significance (*p* = 0.177). There were no significant differences in superficial infection or poor wound healing between the 2 groups (Table 3).

The incidence of reoperation was also significantly higher in the salvage THA group (10.5%, 11/105) than in the primary THA group (3.8%, 8/210) (*p* = 0.019). The results demonstrated that the risks of revision THA were significantly higher in the salvage THA group than in the primary THA group (*p* = 0.003). The curves of surgery-free survival revealed that most revisions occurred within the first 2 years after the index THA. The log-rank test showed significantly worse surgery-free survival in the salvage THA group (*p* = 0.011) (Fig. 1B).

**Fig. 2:**
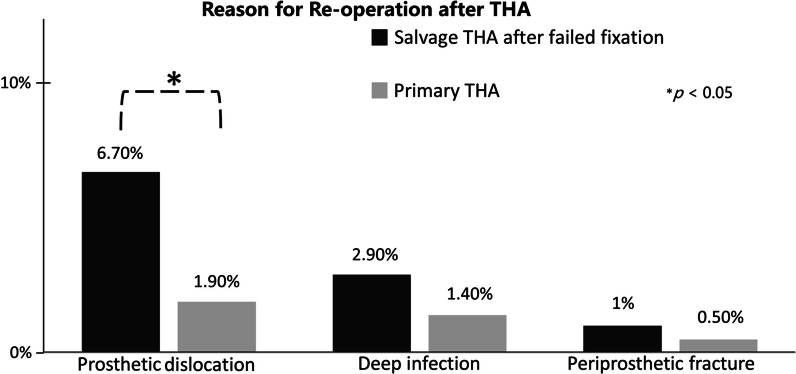
Reason for reoperation after THA

We further analyzed the reason for reoperation. Among the 11 patients (10.5%) who underwent reoperation after salvage THA, 6 had revision surgery involving changing prosthetic components (4 for aseptic loosening after dislocation episodes, 2 for dislocation with concomitant infection). Four patients received open reduction and tightening of the soft tissue envelope for prosthetic dislocations. One patient underwent arthrotomy for debridement. Eight patients (3.8%) received reoperation in the primary THA group, including 4 open reductions for prosthetic dislocations, 3 debridements for deep infections, and one revision surgery with a changing prosthetic stem for periprosthetic fracture. We found that patients who underwent salvage THA had a significantly higher risk of reoperation for dislocation (Fig. 2).

We compared the final Oxford Hip Score to evaluate the joint-specific outcome after total hip arthroplasty. The average Oxford hip scores were 34.8 ± 8 and 36.7 ± 10.4 in the primary THA group and the salvage THA group, respectively (*p* = 0.095). There was no significant difference.

In the subgroup analysis of previous fracture types, patients who had intertrochanteric fractures were older (73.6 ± 11.2 years, *p* < 0.001), more likely to have a longer operative time (149.2 ± 51 min, *p* = 0.032), and more likely to complain of persistent hip pain (83.3%). The incidence of postoperative complications, such as poor wound healing, dislocation, and infection, was also slightly higher, but the difference was not significant (Table 4). On the other hand, patients who had previous fixation for acetabular fracture were younger than the other three groups (48.7 years old, *p* < 0.001), were more likely to be male (73.3%, *p* = 0.025), and had lower risks for persistent hip pain 3 months after the operation. The results of the subgroup analysis based on fixation types were similar (Additional file 1: Table S1).

**Table 4:** Analysis of fracture types of THA after fixation failure

	Previous fracture types			
	Femoral neck fracture	Intertrochanteric fracture	Acetabulum fracture	*p* value
No. of patients	38	37	30	–
Sex (male/female)	17/21	16/21	22/8*	0.025*
Age	63.3 (19.1)	73.6 (11.2)	48.7 (13)	< 0.001*
Operative time (minutes)	118.3 (52.8)	149.2 (51)	129.3 (45.4)	0.032*
Drop in hemoglobin level	2.1 (1.4)	2 (1.4)	2.6 (1.2)	0.15
Blood transfusion	2 (5.3%)	8 (21.6%)	4 (13.3%)	0.114
Amount of blood transfusion	0.1 (0.5)	1.4 (5)	0.4 (1.2)	0.104
Postoperative day to ambulate	2.2 (0.8)	2.2 (1)	2 (0.9)	0.488
Length of stay (day)	7.1 (1.3)	7.6 (1.4)	7.1 (1.3)	0.286
Persistent hip pain	28 (73.7%)	31 (83.8%)	16 (53.3%)	0.022*
Prosthetic dislocation	3 (7.9%)	5 (13.5%)	2 (6.7%)	0.581
Wound poor healing	0 (0%)	3 (8.1%)	0 (0%)	0.059
Superficial infection	0 (0%)	1 (2.7%)	0 (0%)	0.395
Deep infection	0 (0%)	3 (8.1%)	1 (3.3%)	0.184
Reoperation	3 (7.9%)	6 (16.2%)	2 (6.7%)	0.362
THA revision	1 (2.6%)	3 (8.1%)	2 (6.7%)	0.573

Age, operative time, drop in hemoglobin level, postoperative day to ambulate, amount of blood transfusion, and length of stay were expressed as the mean (standard deviation)

Sex, hip pain, prosthetic dislocation, superficial infection, deep infection, poor wound healing, reoperation, readmission, blood transfusion, and THA revision were expressed as numbers (percentages)

Postoperative day to ambulate was defined as walking with a walker for at least 10 m without others' assistance

Persistent hip pain was defined as still having significant hip pain 3 months after THA that needed medication for pain control

*DHS* Dynamic hip screw system

*p < 0.05

## Discussion

The most important findings of this study were that patients undergoing salvage THA after failed internal fixation required more operative time, suffered more blood loss, and had later ambulation. This salvage THA group was also at high risk of early periprosthetic dislocation, periprosthetic infection, reoperation and revision. Most dislocations occurred within 6 months, and most revision THAs were performed within 2 years after the first THA. The subgroup analysis also found that patients with previous intertrochanteric fractures had longer operative times, more postoperative persistent hip pain, and more complications than patients with previous femoral neck fractures and acetabular fractures.

The greater blood loss and longer operative time in the salvage THA group may reflect the blood loss status during the operation. Compared to primary THA, the surgical steps for salvage THA were more complicated, including the additional step of removing previous implants, which may have contributed to more blood loss and longer operative time. In addition, salvage arthroplasty is more technically challenging than primary arthroplasty because the hip is frequently stiff. The adhesive soft tissue and poor bone quality resulted in difficulty in exposure, prolonged operating time, increased blood loss, and increased risk of intraoperative fracture [9].

The postoperative day to ambulate and length of stay may promptly reflect the short-term outcomes after THA. Patients with salvage THA may have impaired progression of the center of pressure and a greater loss of abduction strength in a gait analysis [22]. Our cohort showed that the delay of ambulation was significant. Although a previous study showed a 1.5-day longer hospital stay [25], we did not find a significant difference in our comparison. This was possible because we followed the principles of enhanced recovery after surgery (ERAS) to facilitate recovery after THA [26].

Prosthetic dislocation was one of the most common causes of revision in those who underwent secondary THA after failed fixation. The previous dislocation rate after salvage THA was reported to be 5–19.6% [7, 8, 10, 12, 25, 27]. We found a significantly higher dislocation rate of 9.5% in the salvage THA group than in the primary THA group since poor bone quality in salvage THA causes worse osseointegration. Most of the dislocations occurred within 6 months after salvage surgery. We suggest tightening the soft tissue envelope, repairing the capsule, and reconstructing external rotators during the operation to reduce dislocation risk. Furthermore, emphasis on postoperative hip precaution for at least 3–6 months is important.

A high reoperation rate was reported for THA following internal fixation for proximal femoral fracture [23, 28], with an estimated rate of 18% shown by a recent meta-analysis [28]. Our reoperation rate of salvage THA was 10.5%, but it was also higher than that for primary THA. The most common reason for both reoperation and revision was prosthetic dislocation.

Patients who received salvage arthroplasty after failed internal fixation for fractures had a greater prevalence of complications, leading to a greater need for revision THA [7, 25]. In our study, six patients (5.7%) in the salvage THA group eventually required revision surgery. Three patients underwent revision THA for repeated dislocation, and the other 3 underwent revision THA for deep infection, including 1 patient who had deep infection after open reduction surgery for dislocation. All revision THAs occurred within 2 years after salvage THA. A prior study also showed worse survival outcomes in salvage arthroplasty at both five and ten years [7].

Most previous reports of salvage THA only included patients with proximal femur fractures. We also enrolled 30 (28.6%) salvage THAs after acetabular fracture. These patients were younger and more likely to be male because acetabular fractures are mainly caused by high-energy events [29], and most of our patients were injured in major traffic accidents. After THA, these patients tended to recover well and had less persistent hip pain.

Patients with previous intertrochanteric fractures were the oldest group. Salvage THA in this group required a longer operative time. Elderly patients tended to have a higher incidence of chronic and neuropathic pain [30]. Postoperative pain could exacerbate the condition because of the double crush phenomenon [31]. Therefore, salvage procedures after failed fixation for intertrochanteric fractures might be more problematic [19, 32]. During the follow-up period, persistent hip pain was noted in most patients (83.8%), even 3 months after THA. We also found a trend of more complications, including poor wound healing, infection, and dislocation, in this group.

The limitation of this study is the difficulty in performing a randomized controlled trial for this issue. We designed a 1–2 sex- and age-matched comparison to minimize selection bias. Because this was a single tertiary center cohort, we could analyze the details of comorbidities, types of fracture, survival free from dislocation and revision. All patients followed the clinical pathway in our institution, and the operations were executed by experienced orthopedic surgeons. We focused on the THA revision rate and subgroup analysis, reflecting the actual clinical condition, which has rarely been discussed in the previous literature.

In conclusion, we reported acceptable outcomes of salvage THA after failed internal fixation of fractures. Compared with primary THA, salvage THA requires more operative time, causes more blood loss, delays ambulation, and results in a higher risk of early prosthetic dislocation, reoperation, and revision THA. Subgroup analysis of different etiologies found that patients with previous intertrochanteric fracture might be the most susceptible to postoperative complications.

## Supplementary Information

**Additional file 1: Table S1.** Subgroup analysis of THA after fixation failure.

## Data Availability

The raw data files are available upon request.
