# Supplementary material for: Salvage total hip arthroplasty after failed internal fixation for proximal femur and acetabular fractures
Source: J Orthop Surg Res. 2023 Jan 17;18:45. doi: 10.1186/s13018-023-03519-9 (PMC9843827; doi:10.1186/s13018-023-03519-9)
Supplement: Supplementary file 1 — Additional file 1: Table S1. Subgroup analysis of THA after fixation failure. [file 13018_2023_3519_MOESM1_ESM.docx]

**Supplemental Table 1:** Subgroup analysis of THA after fixation failure

|  |  | **Previous fixation device** | | | |
| --- | --- | --- | --- | --- | --- |
|  | **Screw fixation for femoral neck fracture** | **Cephalomedullary nail fixation for hip fracture** | **DHS or plate fixation for hip fracture** | **Plate fixation for acetabulum fracture** | ***p* value** |
| **No. of patients** | 33 | 20 | 22 | 30 | - |
| **Sex (Male/Female)** | 16/17 | 7/13 | 10/12 | 22/8 | 0.04* |
| **Age** | 61.9 (19.9) | 70.9 (13) | 75.9 (8.3) | 48.7 (13) | <0.001* |
| **Operative time (minutes)** | 116.9 (52.9) | 147.5 (54.1) | 145.8 (50.4) | 129.3 (45.4) | 0.1 |
| **Drop in hemoglobin level** | 2.1 (1.3) | 2 (1.6) | 1.9 (1.4) | 2.6 (1.2) | 0.233 |
| **Blood transfusion** | 2 (6.1%) | 2 (10%) | 10 (21.3%) | 0 (0%) | 0.169 |
| **Amount of blood transfusion (unit)** | 0.1 (0.5) | 0.3 (1) | 2 (6.4) | 0.4 (1.2) | 0.135 |
| **Postoperative day to ambulate** | 2.3 (0.9) | 2.1 (0.8) | 2.3 (1.1) | 2 (0.9) | 0.629 |
| **Length of stay (day)** | 7.2 (1.3) | 7.3 (1.4) | 7.6 (1.3) | 7.1 (1.3) | 0.578 |
| **Persistent hip pain** | 24 (72.7%) | 15 (75%) | 20 (90.9%) | 16 (53.3%) | 0.029* |
| **Prosthetic dislocation** | 3 (9.1%) | 2 (10%) | 3 (13.6%) | 2 (6.7%) | 0.866 |
| **Wound poor healing** | 0 (0%) | 1 (5%) | 2 (9.1%) | 0 (0%) | 0.153 |
| **Superficial infection** | 0 (0%) | 0 (0%) | 1 (4.5%) | 0 (0%) | 0.283 |
| **Deep infection** | 0 (0%) | 1 (5%) | 2 (9.1%) | 1 (3.3%) | 0.38 |
| **Reoperation** | 3 (9.1%) | 3 (15%) | 3 (13.6%) | 2 (6.7%) | 0.752 |
| **THA revision** | 1 (3%) | 2 (10%) | 1 (4.5%) | 2 (6.7%) | 0.746 |

Age, operative time, drop in hemoglobin level, postoperative day to ambulate, amount of blood transfusion, and length of stay were expressed as the mean (standard deviation).

Sex, hip pain, prosthetic dislocation, superficial infection, deep infection, poor wound healing, reoperation, readmission, blood transfusion, and THA revision were expressed as numbers (percentages).

DHS: dynamic hip screw system

Postoperative day to ambulate was defined as walking with a walker for at least 10 meters without others' assistance.

Persistent hip pain was defined as still having significant hip pain 3 months after THA that needed medication for pain control.

**p* < 0.05
